# Stability of Programmable Shunt Valve Settings with Simultaneous Use of the Optune Transducer Array: A Case Report

**DOI:** 10.7759/cureus.675

**Published:** 2016-07-07

**Authors:** Andrew K Chan, Harjus S Birk, Ethan A Winkler, Jennifer A Viner, Jennie W Taylor, Michael W. McDermott

**Affiliations:** 1 Department of Neurological Surgery, University of California, San Francisco; 2 Research Fellow, Howard Hughes Medical Institute; 3 Department of Neuro-oncology, University of California, San Francisco

**Keywords:** shunt valve, hydrocephalus, optune transducer array, glioblastoma multiforme

## Abstract

The Optune® transducer array (Novocure Ltd., Haifa, Israel) is an FDA-approved noninvasive regional therapy that aims to inhibit the growth of glioblastoma multiforme (GBM) cells via utilization of alternating electric fields. Some patients with GBM may develop hydrocephalus and benefit from subsequent shunt placement, but special attention must be paid to patients in whom programmable valves are utilized, given the potential effect of the magnetic fields on valve settings. We present the first case report illustrating the stability of programmable shunt valve settings in a neurosurgical patient undergoing therapy with the Optune device. In this study, shunt valve settings were stable over a period of five days despite Optune therapy. This is reassuring for patients with GBM who require simultaneous treatment with both the Optune device and a programmable shunt system.

## Introduction

The NovoTTF-100A System (Optune TM, Novocure Ltd., Haifa, Israel) is an FDA-approved device that delivers alternating electric tumor treating fields, or TTFields, designed to curtail or prevent division of recurrent glioblastoma multiforme cancer cells. Multiple studies have demonstrated its equivalent efficacy when compared to salvage chemotherapies for recurrent disease [1-2]. Furthermore, studies including the EF-14 Phase III clinical trial have demonstrated the safety of the device without adverse events [1, 3]. Nonetheless, the extent of untoward effects have not been fully characterized.

In a population that may develop hydrocephalus and may require palliative CSF diversion, a potential concern is that the magnetic field may impact programmable shunt valve stability during simultaneous treatment with the Optune device [4]. There are no previous studies assessing the stability of a programmable shunt valve in the context of treatment with alternating electric fields.

Herein, we report the case of a 72-year-old woman who underwent ventriculoperitoneal shunt surgery with the placement of a magnetic programmable shunt valve while undergoing simultaneous treatment with the Optune Transducer Array.

## Case presentation

The patient is a 72-year-old woman with a right temporal glioblastoma multiforme status post temporal craniotomy for resection. She began standard adjuvant fractionated radiation and temozolomide therapy but was notably unable to complete her course of temozolomide due to development of severe myelosuppression. Five months after her resective surgery, the patient began treatment with the NovoTTF-100A Optune System. 

Starting at the patient’s eight-month follow-up visit up through her 11-month follow-up visit, she developed progressive gait ataxia, urinary incontinence, and memory impairment. Magnetic resonance imaging (MRI) of the brain revealed progressive ventriculomegaly concerning for development of hydrocephalus. A lumbar puncture for high-volume CSF removal was undertaken without significant improvement of gait. She was subsequently admitted for a three-day lumbar drain trial. During this hospitalization, no improvements in gait were noted. However, she did display marked improvements in cognition, both with augmented alertness and interaction. Thus, the patient was taken to the operating room for placement of a right frontal ventriculoperitoneal shunt with a Strata® NSC programmable valve (Medtronic, Minnesota, USA) set to 1.0. The patient was discharged on postoperative day 2 without event. 

On the one-month follow-up visit following shunt placement, the patient continued to display persistent improvement in her mental status. However, as a non-contrast head computed tomography (CT) scan revealed development of bilateral subdural hygromas, her Strata valve was changed to level 2.0. It was in this context that the present study was undertaken. 

**Figure 1:**
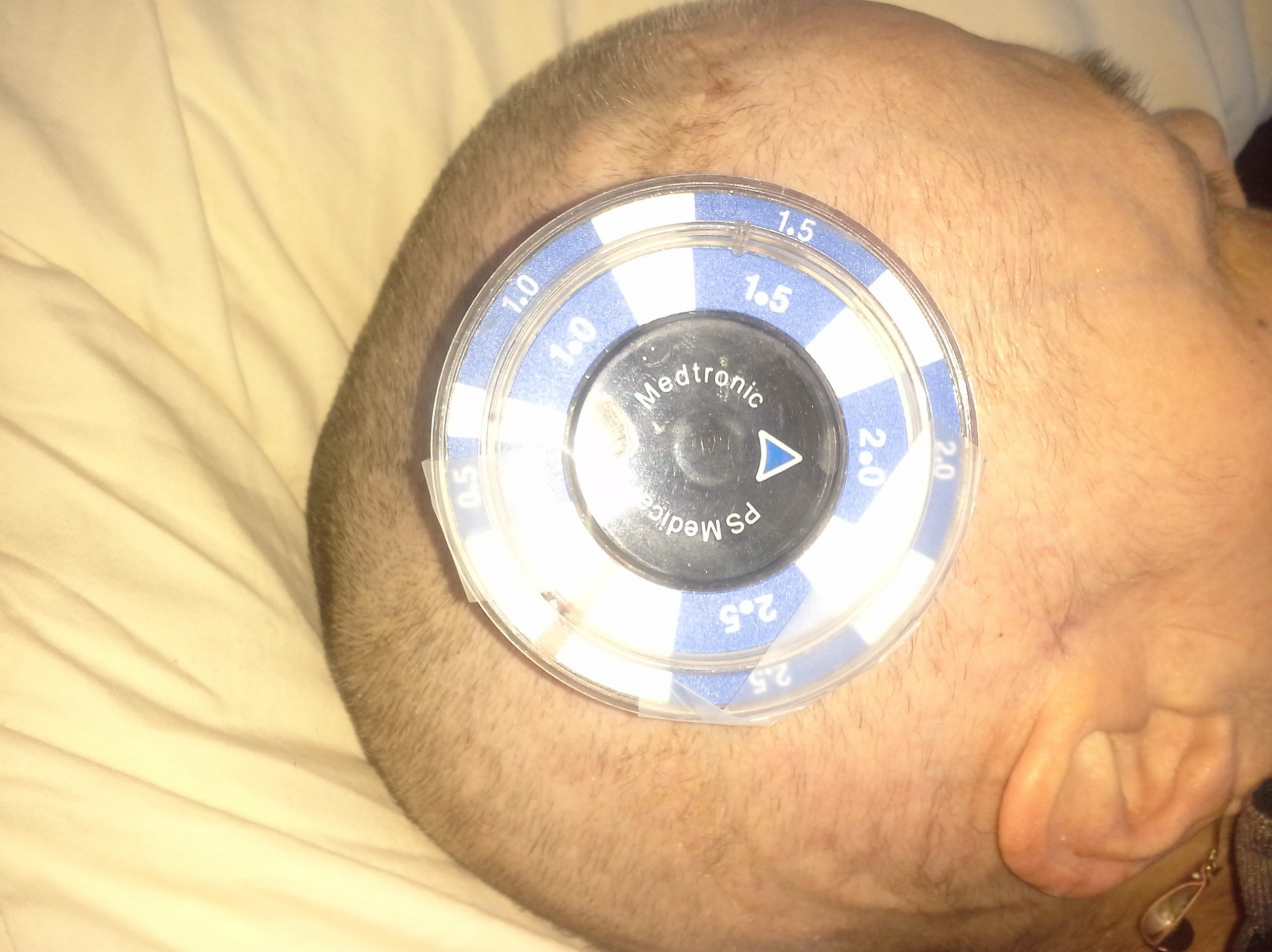
High Resolution Image Revealing Shunt Device Measurement

After written consent was obtained for the study, the patient’s husband was provided with two components of the Medtronic Strata Adjustment Kit: the Locator Tool and the Indicator Tool (Medtronic, Minnesota, USA). The husband was not given the Adjustment Tool. The husband was instructed on the measurement device use by the senior author and attending neurosurgeon of this study. Understanding was confirmed via the teachback method. The husband demonstrated reliable and accurate measurement of the shunt device (Figure 1).

For the next five consecutive days, the setting of the Strata valve was measured with the Optune system–operational and non-operational. All measurements were obtained in the morning with the patient positioned in the left lateral decubitus position. Before the first day of measurements, the location of the Optune gauze was marked with a marking pen, ensuring consistent placement of the Optune.

**Figure 2:**
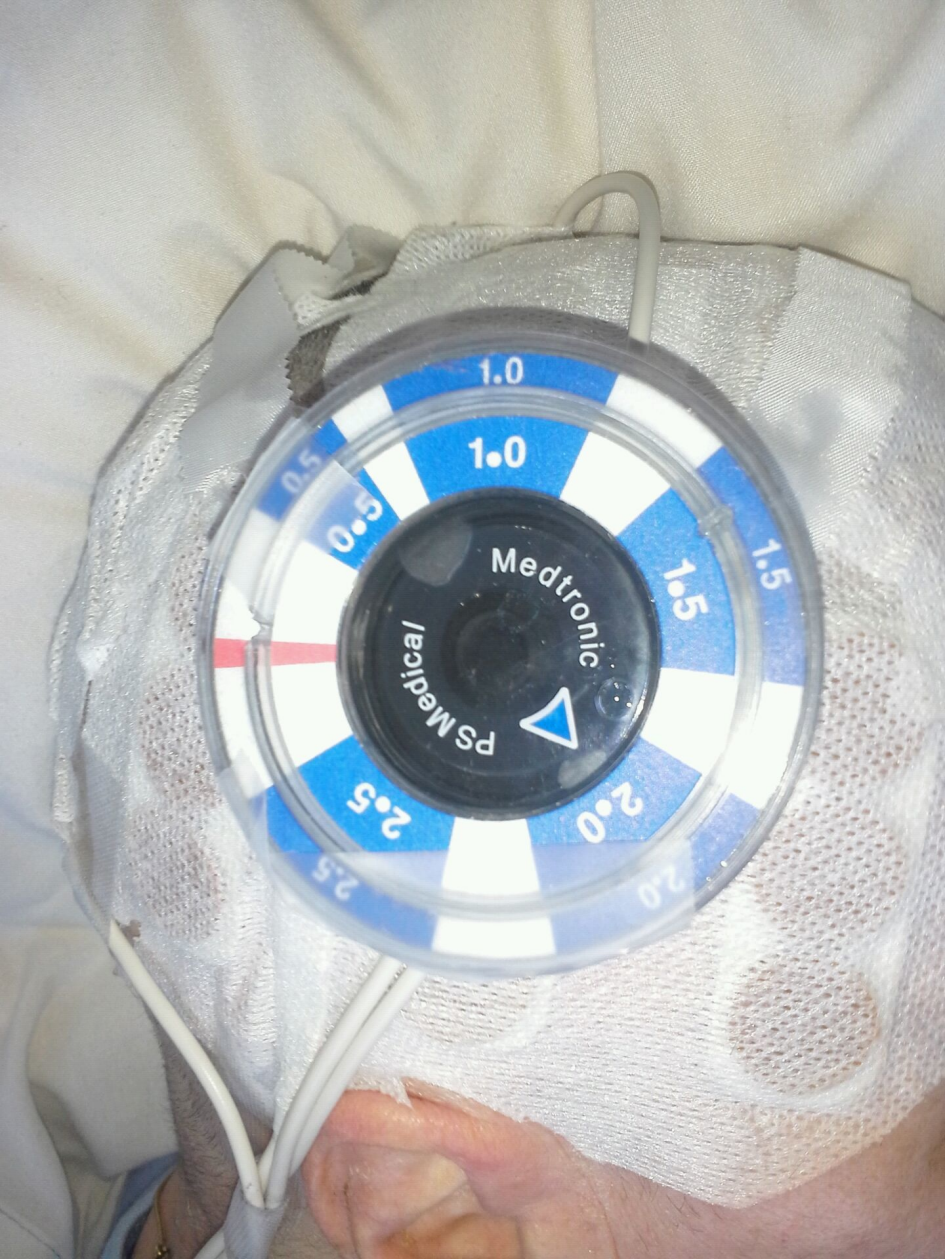
High Resolution Image Showing Optune Array Placement on the Head in Standard Fashion

The patient’s shunt was confirmed by both the husband and author J.A.V. to be at a setting of 2.0 on study day (SD) 1 prior to placement of the Optune system. After initial use of the Optune Transducer Array, which was placed on the head in the standard fashion (Figure 2), the shunt remained at 2.0 (Table 1). The stability of the shunt setting on daily measurements was noted. The patient returned for follow-up on SD 6, where the Strata NSC was confirmed at 2.0 by the senior author (M.W.M.).

**Table 1:** Recording of Shunt Settings as per Daily Measurements

Date	Optune Status	Time	Strata NSC Setting
SD 1	Not yet in place	5:00 PM	2
SD 1	On	8:00 PM	2
SD2	On	7:30 AM	2
SD3	Off	7:30 AM	2
SD4	On	9:00 AM	2
SD4	Off	9:00 AM	2
SD5	On	9:00 AM	2
SD5	Off	9:00 AM	2

At the conclusion of the study, the patient's shunt setting was increased to 2.5 given the persistence of the patient's bilateral subdural hygromas. Of note, the shunt maintained stability at a setting of 2.5 despite concurrent Optune therapy at four-, five-, and 10-week clinic follow-up with measurements confirmed by author J.A.V.

## Discussion

This is the first case report to demonstrate that in a patient undergoing simultaneous therapy with a programmable shunt valve and the Optune transducer array, there is no evidence that the alternating electrical current of the Optune array impacts stability of a programmable, magnetic shunt valve. Optune’s low-intensity electric tumor treating fields are administered through adhesive transducer arrays patches that deliver optimized focalization therapy by enhancing electric field intensity towards the tumor site. The alternating electric fields travel in waves, and are thought to interfere with GBM cancer cell proliferation and repel charged components of tumor cells during mitosis, ultimately curtailing cell growth by focusing solely on the tumor.

Medtronic’s Strata II programmable hydrocephalus shunt is considered magnetic resonance conditional, meaning that although utilization of MRI up to 3.0 Tesla after implantation will not damage the valve, it may alter settings [5]. The recommendation therefore exists that magnetic valve settings be monitored before and after MRI exposure. Given the well-known challenge of maintaining hydrocephalus shunt valve settings in patients undergoing MRI, clinicians and patients may be concerned about the safety of any medical, diagnostic or therapeutic procedure that involves magnetic fields in a patient with a magnetic programmable shunt. However, the relatively localized magnetic field introduced by the Optune differs importantly from that of the magnetic field of an MRI. Patients undergoing therapy with an Optune transducer array experience electric fields that are at highest intensity at the tumor site, thereby decreasing field interference with the magnetic shunt valves [6]. Indeed, we demonstrate that the Optune transducer array does not alter the stability of the Strata NSC shunt setting, likely due to the ability of NovoTTF to generate its manageable alternating electric fields. Furthermore, other issues secondary to the broadly dispersed magnetic field, such as implant overheating and displacement, that arise during simultaneous treatment with programmable valves are less likely to be faced with concurrent utilization of the Optune transducer array because of its highly localized electric fields targeting the tumor site. This localization has been demonstrated by the case study. Attempts to simulate the effects of Optune’s alternating electric fields on a realistic human brain model have revealed that its electric field distributions are nonuniform throughout the brain, generating highest intensity in the gray matter-white matter interface near the tumor, and lowest intensity in the ventricles [7]. 

Per the official Optune patient instruction manual, maximal efficacy is obtained with use of the device for at least 18 hours per day, with treatment breaks recommended only for personal needs such as bathing and exercise. During these times that Optune is switched off, there is no impact on valve settings. In addition, there is a single current setting for the patient such that settings do not require adjustment, enabling a convenient user interface that is conducive towards long-term compliance.

This is the first case report that demonstrates magnetic programmable shunt valve stability on a patient undergoing Optune therapy and it may help to reassure clinicians and patients undergoing concurrent therapy.

## Conclusions

This is the first reported case of a patient with glioblastoma multiforme in whom treatment with the Optune Transducer Array was shown to not alter magnetic programmable shunt settings. This supports the safety of Optune array utilization in neurosurgical patients undergoing simultaneous therapy with programmable shunt-based CSF diversion therapy.
